# Determinants and the Moderating Effects of Individual Characteristics on Autonomous Vehicle Adoption in China

**DOI:** 10.3390/ijerph20010043

**Published:** 2022-12-20

**Authors:** Tianpei Tang, Xiwei Wang, Jianbing Wu, Meining Yuan, Yuntao Guo, Xunqian Xu

**Affiliations:** 1 School of Transportation and Civil Engineering, Nantong University, Nantong 226019, China; 2 Nantong Municipal Engineering Design Institute Co., Ltd., Nantong 226000, China; 3 Department of Traffic Engineering, Key Laboratory of Road and Traffic Engineering, Ministry of Education, Tongji University, Shanghai 201804, China

**Keywords:** autonomous mobility, modified technology acceptance model, policy measure, moderating effects

## Abstract

Along with the increasing popularity of autonomous vehicles (AVs), urban livability and public health will be enhanced due to ecofriendly issues: alleviated traffic congestion, lower car ownership, and reduced transport emissions. However, some emerging issues, including public safety, trust, privacy, reliability, underdeveloped legislation, and liability, may deter user intentions to adopt an AV. This study introduces an extensive factor, playfulness, into the technology acceptance model (TAM) to quantify the impacts of psychological factors (perceived usefulness, perceived ease of use, and perceived playfulness) on AV adoption intention. This study proposes four AV-related policy measures (financial incentivization, information dissemination, convenience, and legal normalization) and examines how policy measures motivate users to adopt an AV to facilitate public safety. Furthermore, this study investigated the moderating effects of demographic factors on the relationships between independent variables and AV adoption intention. Two models were proposed and estimated using a total of 1831 survey responses in China. The psychology-related and policy-related models explained 62.2% and 33.6% of the variance in AV adoption intention, respectively. The results suggest that perceived playfulness (β = 0.524, *p* < 0.001) and information dissemination (β = 0.348, *p* < 0.001) are the most important influencing factors of AV adoption intention. In addition, demographic factors (gender, education, income, the number of private cars owned by a family, and types of cities) can moderate the effects of psychological factors and policy measures on user intentions to adopt an AV. These insights can be employed to design more cost-effective policies and strategies for subgroups of the population to maximize the AV adoption intention.

## 1. Introduction

The autonomous vehicles (AVs) considered in this study are driverless cars that adopt sensing and communication technologies to navigate safely and efficiently without human intervention. They will be an integral part of a future intelligent transportation system that will require a coordinated awareness of the traffic environment, planning decisions, and multilevel assisted driving to ensure public safety. Research into AVs and supporting infrastructure, such as the 5G cellular network, has recently made rapid progress, and the subsequent development of AVs has led to their increased commercialization. Some vehicle manufacturers (e.g., BMW and Volvo) project to incorporate autonomous driving features in their traditional vehicles by 2040 [1]. By 2040, AVs are expected to represent 25% of the global market for private vehicles, according to previous studies [2]. Thus, AVs have become a high-potential alternative to positively promote public safety and a promising traffic environment.

A wide range of societal and individual benefits will result from the adoption of AVs in the private vehicle market, including enhanced traffic safety, urban livability, and a better user experience [2,3,4,5,6,7]. Increased AV use would reduce vehicle collisions by 90% because more than 90% of traffic accidents are caused by driver errors and misjudgments [3]. Urban livability and public health will be enhanced due to alleviated traffic congestion, lower car ownership, reduced needs for transportation infrastructure (e.g., parking spaces), and reduced transport emissions. Massar et al. [6] revealed that the highest emissions reduction might occur between 60 and 80% AV penetration into the daily mobility, while the expected emissions reduction might not be realized at a lower rate of AV penetration. The use of AVs will enable users to complete more productive tasks during traveling and will provide mobility to people who would otherwise be restricted in travel (e.g., the elderly and the disabled). However, Haboucha et al. [8] found that the use of AVs was met with significant popular opposition due to the perception of there being a significant technological leap from the use of traditional vehicles. In 2020, the Ministry of Transport of China issued guidelines on promoting the development and application of autonomous driving technology, including enhancing the research of autonomous driving technology, improving the intelligence level of road infrastructure, promoting the pilot application of autonomous driving technology, and designing the risk prevention and control system. However, the application of autonomous driving technology is still very limited. AVs will not realize their benefits if potential users do not adopt them because of perceived drawbacks. Potential users are expected to switch to AVs rather than drive themselves, so their perspectives on AVs and the factors that influence their intentions to adopt AVs merit examination.

Extensive studies have adopted the technology acceptance model (TAM) with extensive factors (trust, relative advantage, and privacy concerns) to identify the psychological factors (e.g., perceived usefulness and perceived ease of use) that influence individual intentions to adopt an AV [9,10,11,12]. However, some noteworthy factors, such as perceived playfulness, were not introduced or examined. Despite many studies of intentions to adopt AVs, little is known about how policy measures influence attitudes towards AV adoption. Many efforts were made and many policies were introduced to incentivize electric vehicle (EV) use in the early stage of EV development. These efforts were mainly to subsidize EV buyers, construct charging facilities, provide accurate information related to EV performance, and construct dedicated parking spaces for EVs [13,14,15,16]. Many researchers have examined the effects of financial incentivization on intentions to adopt EVs [13,17,18]. Wang et al. [19] additionally considered two policy measures (the provision of information and raising the awareness of user convenience) and investigated the effects of these policies on intentions to adopt EVs to provide more insights into EV adoption. These policies directed towards EV promotion might be similarly applied in promoting AV adoption. In the future mixed-traffic environment of driven and driverless vehicles, the use of AVs may introduce legal issues [20,21]. For example, the liability for a traffic accident is difficult to define when an AV crashes with another road user or pedestrian. To address these legal issues, the laws, insurance, and oversight related to AVs should be strengthened prior to the widespread use of AVs. Thus, legal review and reform should be a fourth policy measure to promote AV adoption. In this study, financial incentivization was intended to decrease the costs of purchasing and operating an AV and included policies such as direct subsidies, tax exemptions, and road toll exemptions. Policies of AV information dissemination included information about the practicality, reliability, safety, ease of use, fuel consumption, and environmental performance of AVs, among other issues. Policies concerned with the convenience of AV use included dedicated road lanes, higher speed limits, and the removal of restrictions such as even and odd license plate number rules. Legal review and reform policies were intended to provide a supportive legal environment for users of AVs and included measures such as improving the application of laws, insurance, and governance to AVs. We refer to this as the legal normalization of AVs. In addition, user demographic features also play a significant impact because they can demonstrate differences in demographics on user intentions to adopt an AV. Previous studies have considered demographic variables as control variables [8,22,23], but little work has been conducted on their moderating effects on influencing factors related to adoption intention.

The purpose of this study was to (1) examine the influences of TAM-related psychological factors and policy measures on AV adoption intention and (2) explore the moderating effects of demographic variables on the relationships between independent variables and AV adoption intention. Our study contributes to the research literature by providing an increased understanding of individual preferences in AV adoption. The empirical evidence gathered in this research has practical implications for the future wider adoption of AVs in China.

## 2. Literature Review and Hypothesis Development

### 2.1. TAM-Related Factors

TAM was developed by Davis [24] to investigate the key factors influencing the use or non-use of a new technology, information system, or other innovation. TAM is a sound approach to proactive exploration in the development stages of a user-related intention to adopt a technology [25,26,27]. TAM has been employed to study the use or adoption of AVs. Koul and Eydgahi [28] adopted the original TAM to explore the potential adoption of AV technology. Several studies have extended TAM by incorporating a range of psychological factors, such as trust, relative advantage, privacy concerns, subjective norms, and personal innovation, to reach a better understanding of AV acceptance [2,9,10,12,29,30,31,32,33,34]. These studies demonstrated that TAM is a sound theoretical model that can include many parameters that affect individual intentions to adopt an AV.

The original TAM included two particular constructs: perceived usefulness (PU) and perceived ease of use (PEU). PU represents the extent to which a user believes that an AV will improve their work performance. PEU indicates a user’s perception of the effort required to use an AV. An additional parameter, adoption intention (AI), indicates user readiness to adopt an AV. Causal relationships among these three constructs have been demonstrated in different technologies, such as handheld internet devices [35], mobile chat services [36], mobile commerce [37], and autonomous driving [9,32,33,34]. The following are the hypotheses concerning these factors:

**H1.**  *A greater PU indicates a greater intention to adopt an AV.*

**H2.**  *A greater PEU indicates a greater intention to adopt an AV.*

Recent studies showed that playfulness has been increasingly interpreted as an interactive characteristic that indicates fun and cognitive immersion [38,39]. The researchers cited introduced playfulness as an additional construct to indicate the enjoyment gained from using a mobile app within the theoretical framework developed by Moon and Kim [40], who first used playfulness as a construct. Moon and Kim [40] concluded that if an IT system did not provide user enjoyment, willingness to use it decreased. Previous studies have shown that greater perceived playfulness (PP) indicates an increased intention to use a new technology. Few studies have considered PP as a factor to investigate user intentions to adopt an AV. PP indicates the extent to which a potential user of an AV finds an AV to be fun or interesting. In this study, we attempted to verify the importance of PP using hypothesis H3, as we think that PP as well as PU and PEU might increase individual intentions to adopt an AV.

**H3.**  *A greater PP indicates a greater intention to adopt an AV.*

### 2.2. Policy Measures

This study incorporated perceptions related to four policies: financial incentivization (FI), information dissemination (ID), convenience (CO), and legal normalization (LN) to better understand individual intentions to adopt an AV.

#### 2.2.1. Financial Incentivization

An AV costs significantly more than a conventional automobile because it is an innovative technology. In a similar technology introduction, early adopters of EVs were concerned about the purchase cost and viewed a competitive cost as a decision factor [17,41]. A high purchase cost was a key impediment to EV adoption [17,42,43]. Different countries produced different financial incentivizations to promote EV uptake. For example, before 2015 the Chinese government stated that it would provide a subsidy of up to CNY 60,000 (about USD 9500) to the purchaser of an EV; the amounts of the subsidies for EVs depended on the vehicle technology, vehicle category, and vehicle efficiency [44] The United States government introduced rebate programs to encourage EV uptake [45]. The Norwegian government abolished the purchase tax, value-added tax, and road tolls on EVs [46]. A new vehicle registration tax was decreased in Ireland to stimulate consumer purchases of EVs [47]. It is evident that financial incentivization lowered the cost of EV adoption, thereby increasing individual intentions to adopt an EV [15,17,18,19,48]. Shabanpour et al. [49] and Chen et al. [50] found that AV purchase prices affected user choice in AV uptake. It is reasonable to suppose that increased financial incentivization can increase user intentions to adopt an AV. Therefore, the following hypothesis was framed:

**H4.**  *FI has a positive effect on user intentions to adopt an AV.*

#### 2.2.2. Information Dissemination

Previous studies have shown that product information can motivate the sales of a product [51,52,53]. For example, in the context of automobile consumption, Graham-Rowe et al. [53] revealed that information about vehicles significantly affected consumer purchasing decisions. AVs represent an innovative vehicle technology in its infancy, and information about them is almost unknown or misunderstood by potential users; this may negatively affect their intentions to adopt an AV. Drawing on the early uptake of EVs for similarity, most potential users were inclined to desist from EV purchase or to take a wait-and-see attitude, as they had little knowledge of EV performance (e.g., safety, reliability, and range) [42,54,55]. Several studies found that providing additional details about EVs, including the reliability, safety, range per charge, battery life, charging time, and environmental performance, among other issues, to users familiarized them with EVs and made them more inclined to adopt an EV [14,18,42,54]. Therefore, if government agencies take steps to increase the availability of AV information (e.g., the safety, reliability, travel efficiency, and environmental protection), users might be more inclined to adopt an AV, as formulated in hypothesis H5.

**H5.**  *ID has a positive effect on user intentions to adopt an AV.*

#### 2.2.3. Convenience

Previous studies of public transportation have shown that giving priority to transit vehicles (e.g., dedicated bus lanes and rights of way) is an effective way to promote the use of public transportation and to promote sustainable urban development [56,57]. Several studies of EVs found that charging difficulty, long charging times, and limited driving range were the three major barriers that led consumers to believe it was inconvenient to adopt an EV [19,45,55,58]. Several policies to facilitate the use of EVs by making them more convenient have been developed and implemented. For example, in China, EVs are not bound by the rules of even and odd license plate numbers in some cities (e.g., Beijing) [43], and EVs are allowed to drive in dedicated bus lanes and have dedicated parking zones [43]. A policy of no traffic limitations on EVs greatly affected user attitudes towards adopting EVs [58]. Varian [59] concluded that consumers intended to adopt new items if the cost was less than the anticipated returns and benefits. In essence, the convenience can be regarded as an added bonus for consumers. In previous studies of AVs, Shabanpour et al. [49] and Chen et al. [50] found that the provision of exclusive lanes for AVs affected user choice. Thus, a policy of providing more convenience could increase AV uptake, which we formulated as hypothesis H6.

**H6.**  *CO has a positive effect on user intentions to adopt an AV.*

#### 2.2.4. Legal Normalization

AV technology has the potential to reduce traffic fatalities while also providing elderly and disabled people with independent and accessible mobility. However, it raises some legal issues [20,21]. For example, if a fully AV collides with a conventional vehicle driven by a person and kills a pedestrian strolling nearby, who is to blame? When the conventional vehicle’s driver is found to be not at fault, this question becomes much more challenging to answer. Is the AV manufacturer subject to both civil and criminal liability? Should the low level of foreseeability of the incident be considered? Is it possible to hold an AV or its head (the device or artificial intelligence (AI) that controlled it) responsible for the outcome? This sort of problem, especially one in which no human entity can be ascribed liability, is a topic of considerable interest around the world. AV users might have concerns over such potential disadvantages caused by similar legal issues and refuse to adopt AVs. Thus, normalizing the legal doctrine (e.g., laws, insurance, and supervision related to AVs) may promote AV adoption. We formalized these ideas in hypothesis H7.

**H7.**  *LN has a positive effect on user intentions to adopt an AV.*

### 2.3. Demographic Factors

Previous studies revealed that some demographic factors, including gender, age, education, personal income, and the number of private cars owned by a family, have significant effects on AV adoption intention [2,9,10,12,29,30,31,32,33,34]. However, few studies of AVs have explicitly investigated the moderating effects of demographic factors on the effects of psychological factors or policy measures on AV adoption intention. It is worth noting that no literature has discussed the impact of the types of cities on AV adoption intention. Our work was intended to fill this research gap. We considered gender, age, education, personal income, the number of private cars owned by a family, and the types of cities to be control variables in this study. Demographic variables might moderate the relationships between independent factors and adoption intention. We proposed the following hypotheses to formulate these views.

**H8**–**H13.**  *The demographic variables gender (H8), age (H9), education (H10), personal income (H11), the number of private cars owned by a family (H12), and the types of cities (H13) can moderate the effects of (a) PU, (b) PEU, (c) PP, (d) FI, (e) ID, (f) CO, and (g) LN on AV adoption intention.*

### 2.4. Research Framework

**Figure 1:**
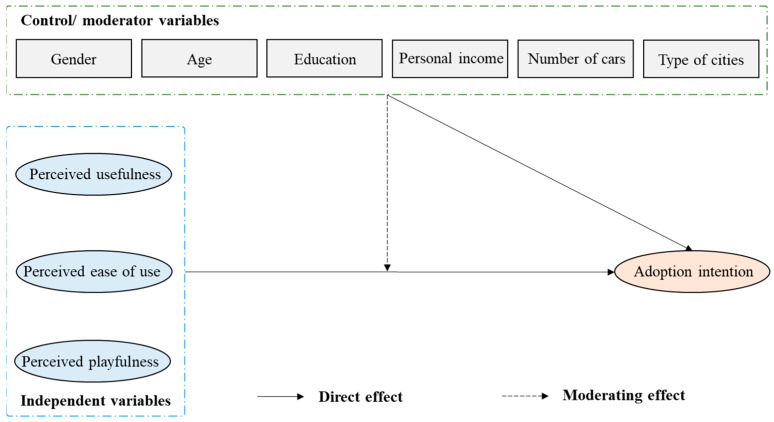
Research framework of relationships among psychological factors, demographic factors, and AV adoption intention.

**Figure 2:**
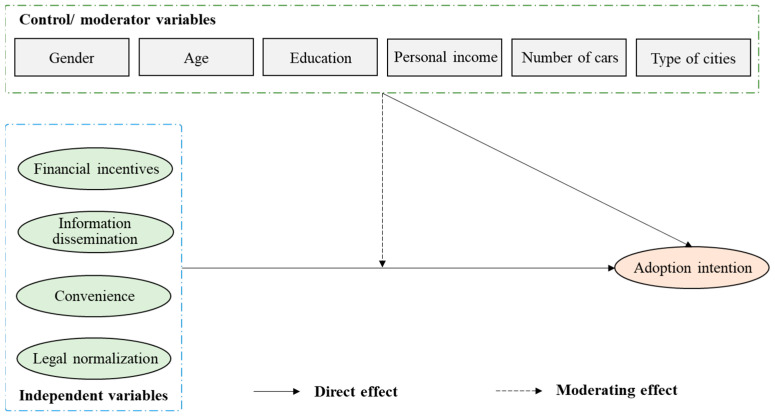
Research framework of relationships among policy measures, demographic factors, and AV adoption intention.

In this study, we focused on three psychological factors (PU, PEU, and PP), four policy measures (FI, ID, CO, and LN), and six demographic factors to develop two research frameworks for investigating user intention to adopt an AV (as shown in Figure 1 and Figure 2). In these frameworks, the moderating effects mean that the demographic factors promote or inhibit the relationships between independent factors and AV adoption intention.

## 3. Methodology

### 3.1. Measurement

The aforementioned constructs were measured by multiple items. PU, PEU, and adoption intention were measured by three items that were modified from other studies [32,34,39]. Three items were modified from the studies by Ahn et al. [38] and Hur et al. [39] to measure PP. Each policy measure was measured by three items. Measures for FI, ID, and CO were adapted from previous studies of EVs or AVs [19,49,50,58]. LN measures were developed using previous studies on the legal regulation of AVs [20,21]. A five-point Likert scale (from 1 (strongly disagree) to 5 (strongly agree)) was used to score each item. A list of these measuring items is provided in the Appendix A (Table A1).

A questionnaire was developed that consisted of three sections: the introduction of AVs to the participants, the demographic features of the participants, and the measurement items. In the first part, an introduction to AVs was given to the participants based on the Taxonomy of Driving Automation for Vehicles (2021) in China for AVs with level 5 automation: “Self-driving cars can perform all driving tasks—essentially, do all the driving. You do not need to take over driving in those conditions”. This taxonomy was localized based on the SAE (Society of Automotive Engineers) levels of driving automation, and the classification system is mainly the same. It is important to note that participants needed to share a consistent understanding of the definition of Avs, as the understanding of the term can vary greatly between individuals. A pilot survey was first conducted among 50 participants to ensure that the questionnaire was understandable and could be completed within a reasonable time. The finalized questionnaire was modified according to the incomprehensible items reported by the participants.

### 3.2. Sampling and Data Collection

The questionnaire survey was administered to obtain data for testing the research hypotheses. Data for the study were gathered by Sojump Survey Company from March to April of 2021. Sojump Survey Company is one of the largest survey platforms in China and has been employed extensively to carry out transportation-related studies in China [15,16,18,19]. To cover more potential users in China, about 2000 online questionnaires were distributed to residents in different developed cities (first-, second-, and third-tier cities). The survey had five attention-check questions in which participants had to choose a specific response. Only responses that successfully completed the attention-check questions were considered complete and valid. All participants were rewarded with CNY 5 (approximately USD 0.76) for finishing the survey. In all, 1831 complete and valid responses were collected. The participants’ IP addresses showed that the valid questionnaires were from first-tier cities (e.g., Beijing, Shanghai, and Suzhou; n = 522), second-tier cities (Ningbo, Nantong, and Lanzhou; n = 447), and third-tier cities (Weihai, Wuhu, and Xinxiang; n = 862). These city tiers are based on the population and GDP.

The demographic characteristics of the 1831 survey participants are shown in Table 1. Among the participants, 53.3% were female. Nearly half of the participants (45.6%) were 18–24 years old, and about one third (32.2%) were 25–39 years old. They were well educated, and 69.2% of them had bachelor’s degrees or above; those who graduated senior high school, polytechnic school, or below accounted for 11.1% of the respondents. Most respondents had annual personal incomes in the ranges of CNY 50,000–100,000 (36.2%) or CNY 100,001–200,000 (36.9%). In total, 69.8% of households owned a private car, and 21.5% of them owned two or more private cars.

### 3.3. Data Analysis

Reliability and validity tests were conducted to evaluate the internal consistency and validity of the measurement instrument. A value of 0.6 was adopted as the cutoff factor loading based on Nunnally and Bernstein [60], and any items lower than that were not included. Cronbach’s α is used to test how closely related a set of items were as a group. A Cronbach’s α value exceeding 0.7 was considered good [61]. Composite reliability (CR) measures the degree to which observed variables account for latent variables, which should be greater than 0.70 [62]. The convergent validity of the items was evaluated using the average variance extracted (AVE) values, which should exceed 0.50 [62]. Discriminant validity was evaluated to determine whether theoretically distinct concepts were also empirically distinct. Thus, the square roots of the AVEs were inspected [61]. The square roots of the AVEs of each construct should be greater than the correlations among constructs.

To test the hypothesized relationships, a multiple linear regression analysis was used to estimate the influences of psychological factors and policy measures on AV adoption intention as well as to estimate the moderating effects of demographic variables on the relationships between these independent factors and AV adoption intention. The independent and moderator variables were mean-centered before the regression analysis. The statistical analyses were conducted using AMOS 24.0 and SPSS Statistics 24.0.

## 4. Data Analysis and Results

### 4.1. Reliability and Validity Tests

Table 2 and Table 3 display the results of the reliability and validity tests. PU2, PP3, ID1, and CO2 were removed because the factor loadings of these items were <0.6. The factor loadings of the remaining items were in the range of 0.620–0.773 and were significant at *p* < 0.001 (Table 2). In the reliability tests, the CR values of the constructs were in the range of 0.702–0.796, and Cronbach’s α values were in the range of 0.702–0.796 (Table 2). These values were higher than the suggested threshold value of 0.7 and indicated the high internal consistency of the scales used in this study. In convergent validity testing, the AVE values of all constructs are in the range of 0.501–0.566 and were above the benchmark value 0.50 (Table 2). In the discriminant validity test, the square roots of the AVEs were greater than the correlations between the constructs (Table 3). In sum, the reliability and validity of these constructs were satisfactory.

### 4.2. Hypothesis Testing

A multiple linear regression analysis was conducted to examine the hypothetical relationships between the variables. The impacts of the psychological factors and the moderating effects of the demographic factors are shown in Table 4. Model 1 was a basic model that was created by introducing the control variables. Model 2 was based on the original TAM, and model 3 extended it by introducing PP. Model 4 additionally factored in the moderating effects of the demographic variables. In the final model (model 4), PU (β = 0.278, *p* < 0.001), PEU (β = 0.141, *p* < 0.001), and PP (β = 0.524, *p* < 0.001) had significant positive effects on AV adoption intention, which supports hypotheses H1, H2, and H3. PP had the strongest effect, while PEU had the weakest effect. Based on the models’ goodness of fit results, we also noted that incorporating PP can considerably increase the explanatory utility of the model. Furthermore, the findings display that gender had significant negative moderating effects on the relationships between PU (β = −0.125, *p* < 0.01), PP (β = −0.117, *p* < 0.01), and adoption intention; education could significantly and positively moderate the relationship between PP and adoption intention (β = 0.082, *p* < 0.01); and income had a moderating effect on the relationship between PU and adoption intention, which was significant and positive (β = 0.056, *p* < 0.05). No moderating effects of age, the number of private cars owned by a family, or the types of cities were observed on the relationships between the psychological factors and adoption intention. Thus, Hypotheses H8a, H8c, H10c, and H11a were supported, but the others were not.

Table 5 shows the impacts of the policy measures and the moderating effects of the demographic factors. Model 1 was a basic model and model 2 extended it by introducing four policy-related variables. Model 3 additionally considered the moderating effects of the demographic variables. In the final model (model 3), the path coefficients from FI, ID, CO, and LN (β = 0.216, *p* < 0.001; β = 0.348, *p* < 0.001; β = 0.121, *p* < 0.001; β = 0.113, *p* < 0.001) were significant and positive. These findings support hypotheses H4, H5, H6, and H7. The importance of the four policy measures was ordered (high–low) ID, FI, CO, and LN. Furthermore, the findings revealed that education had a significant positive moderating effect on the relationship between LN and adoption intention (β = 0.092, *p* < 0.01); the moderating effect of income on the relationship between FI and adoption intention was negative and significant (β = −0.053, *p* < 0.05); the number of private cars owned by a family could significantly and negatively moderate the relationship between ID and adoption intention (β = −0.065, *p* < 0.05); and the types of cities had a significant moderating effect on the relationship between FI (β = 0.053, *p* < 0.05), CO (β = −0.069, *p* < 0.05), and adoption intention. Gender and age had no significant moderating effects on the relationships between policy measures and adoption intention. As such, hypotheses H10g, H11d, H12e, H13d, and H13f were supported, but the others were not.

## 5. Discussion

The findings of this study show that all TAM-related factors and policy measures significantly influenced the adoption intention of potential Chinese AV users and that several demographic factors could moderate the influences of these independent factors on adoption intention.

Among the three psychological factors, PU had a stronger impact on adoption intention than PEU, which is consistent with previous studies of AVs [9,32,33,34]. The effect of PP has rarely been investigated in AV research. We observed that PP showed the strongest effect on AV adoption intention, which indicates that PP is the most important factor in user decision making. A plausible reason is that users are more motivated to purchase a new technological product (e.g., a foldable smartphone or a wearable VR/AR) by the playfulness it engenders rather than its usefulness or ease of use. Thus, if users perceive that many features of AVs are interesting and using AVs is fun, they will be more likely to adopt an AV. Furthermore, male users will be more strongly impacted by PU and PP in their intentions to adopt an AV than female users because they might favor something that will boost their productivity and entertainment. The effect of PP on AV adoption intention was also influenced by education. Potential users who have higher education levels had a significantly greater impact of PP on the intention to adopt an AV than those who had lower education levels. The reasons might be that users with higher education levels have a better pursuit of life and travel quality, hoping to obtain more enjoyment from various social activities. Higher-income users had a significantly greater impact of PU on adoption intention than lower-income users. One plausible reason is that higher-income users usually pursue more economic efficiency and prefer some instrumental products that can deliver real benefits.

Among the four policy measures, ID had the greatest influence on adoption intention. AVs are vehicles that embody innovative technology and are in their infancy. These findings emphasize the value of adopting informational campaigns to encourage users’ understanding of novel technologies’ capabilities and limitations in their adoption process. This is because many early users of new technologies actively seek out information pertaining to the new technologies’ anticipated characteristics as well as any advantages or disadvantages related to their use. Information about their many attributes may be unknown or misunderstood by potential users, and this may negatively influence adoption intention. Some studies have found that many potential users are inclined to resist AVs or have a wait-and-see attitude due to emerging issues (e.g., trust, privacy, and reliability) around AVs [8,63,64]. Therefore, providing more information may motivate potential users to switch from resisting AVs to acquiring them. FI was the second most important factor. As more financial incentivization is provided, user intentions to adopt an AV are strengthened, which is consistent with previous studies [49,50]. The third most important factor was CO. The results suggest that providing dedicated lanes, allowing higher speed limits for Avs, and lifting restrictions of the rules of even and odd license plate numbers for AVs would attractive more users to adopt AVs. The weakest influencing factor was LN. This means that, although legal issues related to AVs are a hot topic in academia and industry [20,21], users pay little attention to them in the infancy of AV uptake, so LN has a weak impact on adoption intention. The influence of LN on AV adoption intention might become more topical in the future when AV users are confronted with significant legal issues. Furthermore, more highly educated users will be more greatly impacted by LN on their adoption intention because they will be aware of the legal risks associated with using AVs and will pay more attention to legal issues related to AVs. Expectedly, lower-income users had a significantly greater impact of FI on adoption intention than higher-income users, as they are more price sensitive to products. This also explains why potential users who live in lower-tier cities had a greater impact of FI on adoption intention than those who live in higher-tier cities. Additionally, the influence of CO on adoption intention was stronger among the higher-tier city users because these cities are well developed and can provide more infrastructure to promote the commercialization of AVs. Interestingly, this study revealed that the potential users who have less private cars will be more greatly influenced by ID in their intention to adopt AVs. One plausible reason is that they have less awareness of car-related information; hence, providing more positive information about AVs might be more beneficial to encourage them to adopt AVs.

## 6. Conclusions 

Our study reveals how psychological factors and policy measures influence Chinese users to adopt AVs and shows how demographic factors moderate these relationships. Three important theoretical conclusions and some managerial applications are provided to motivate AV safety and ecofriendly development:

(1) The present study extended the TAM model by introducing an extensive factor, PP, when examining the psychological factors that might influence AV adoption intention. The findings revealed that the importance of the three psychological factors is ordered (high–low) PP (β = 0.524, *p* < 0.001), PU (β = 0.278, *p* < 0.001), and PEU (β = 0.141, *p* < 0.001). This ranking shows that early adopters of AVs are concerned most with the enjoyment gained from using an AV. Therefore, to facilitate AV safety and maturing, in addition to improving the functionality and technology of AVs, manufacturers and AV developers are advised to develop more in-vehicle entertainment features, which might make them more attractive to potential adopters.

(2) Different policy measures have different influences on AV uptake. We identified four policy areas, including finance, information, convenience, and legislation, in which policy can influence adoption. The importance of the four policy measures is ordered (high–low) ID (β = 0.348, *p* < 0.001), FI (β = 0.216, *p* < 0.001), CO (β = 0.121, *p* < 0.001), and LN (β = 0.113, *p* < 0.001). Thus, the government should put more effort into providing information related to the benefits of AVs (e.g., the practicality, reliability, safety, ease of use, fuel consumption, and environmental performance) to promote the early adoption of AVs.

(3) Our study investigated the moderating effects of demographic factors on the relationships between independent variables and AV adoption intention. Several demographic variables (gender, education, income, the number of private cars owned by a family, and the types of cities) showed differences in the influences of psychological factors and policy measures on AV adoption intention. Therefore, policymakers should tailor interventions and policies to potential AV users with different demographic characteristics. In the future, the above psychological investigation findings may be used to significantly promote AV development.

## 7. Limitations

This study revealed several interesting findings and implications, but it included the following limitations: The sample was likely skewed due to participants’ prior experience with AVs. Nordhoff et al. [65] indicated that the lack of user experience limited an AV acceptance study. Thus, our sample was particularly crucial for researching subjects who expressed an intention to adopt an AV, and the results were obtained from participants with AV consumer experience. In our next study, we will focus on collecting questionnaire data from participants who have adopted an AV. Second, the policy measures (external factors) may promote or inhibit the influences of users’ psychological factors (internal factors) on their intentions to adopt an AV. Future studies will focus on the moderating effects of policy measures. Finally, the influences of psychological factors and policy measures may change as AV technology changes and becomes better commercialized. Dynamic studies are required to understand this process.

## Figures and Tables

**Table 1:** Demographic characteristics of participants.

Demographics	Frequency	Percentage (%)
Gender		
Male	855	46.7
Female	976	53.3
Age		
<18	66	3.6
18–24	837	45.6
25–39	589	32.2
40–59	248	13.6
≥60	91	5.0
Education		
Senior high school/polytechnic school or below	203	11.1
Associate degree	360	19.7
Bachelor’s degree	1140	62.2
Master’s degree or above	128	7.0
Personal income (net annually)		
≤CNY 50,000 (USD 7937)	308	16.8
CNY 50,000–CNY 100,000 (USD 7937–USD 15,873)	663	36.2
CNY 100,001–CNY 200,000 (USD 15,873–USD 31,746)	676	36.9
> CNY 200,000 (USD 31,746)	184	10.1
Number of private cars owned by a family		
0	160	8.7
1	1278	69.8
2	335	18.3
>2	58	3.2
Types of cities		
First-tier cities	522	28.5
Second-tier cities	447	24.4
Third-tier cities	862	47.1

**Table 2:** Reliability and validity tests of the constructs.

Constructs	Items	Mean	SD	Cronbach’s α	Standardized Factor Loading	CR	AVE
PU	PU1	3.68	0.95	0.702	0.725	0.704	0.543
	PU2 ^a^	-	-		-		
	PU3	3.88	0.98		0.749		
PEU	PEU1	3.65	1.04	0.728	0.738	0.750	0.502
	PEU2	3.97	1.00		0.760		
	PEU3	4.11	0.92		0.620		
PP	PP1	3.77	0.97	0.720	0.730	0.720	0.563
	PP2	3.92	0.99		0.770		
	PP3 ^a^	-	-		-		
FI	FI1	4.14	0.89	0.737	0.676	0.763	0.519
	FI2	3.76	1.03		0.697		
	FI3	4.05	0.95		0.783		
ID	ID1 ^a^	-	-		-		
	ID2	3.81	0.96	0.712	0.697	0.702	0.542
	ID3	3.80	0.98		0.773		
CO	CO1	4.03	0.98	0.704	0.723	0.710	0.551
	CO2 ^a^	-	-		-		
	CO3	3.86	1.08		0.761		
LN	LN1	4.34	0.83	0.750	0.706	0.751	0.501
	LN2	4.29	0.86		0.727		
	LN3	4.28	0.85		0.690		
AI	AI1	3.80	1.00	0.796	0.768	0.796	0.566
	AI2	4.00	1.01		0.714		
	AI3	3.62	1.10		0.773		

Note: SD is short for standard deviation. ^a^ Factor removed due to reliability concerns (standardized loading less than 0.60).

**Table 3:** Means, standard deviations, and correlations.

Constructs	Means	SD	PU	PEU	PP	FI	ID	CO	LN	AI
PU	3.78	0.84	**0.737**							
PEU	3.91	0.80	0.466 **	**0.709**						
PP	3.84	0.87	0.635 ***	0.453 **	**0.750**					
FI	3.98	0.78	0.399 **	0.272 *	0.375 **	**0.720**				
ID	3.81	0.83	0.491 ***	0.305 **	0.475 **	0.437 ***	**0.736**			
CO	3.94	0.83	0.426 **	0.308 **	0.399 **	0.503 ***	0.417 **	**0.742**		
LN	4.30	0.69	0.352 **	0.298 *	0.370 **	0.378 **	0.413 **	0.419 **	**0.708**	
AI	3.81	0.88	0.649 ***	0.484 **	0.636 ***	0.419 **	0.499 ***	0.384 **	0.348 **	**0.752**

Note: N = 446. *** *p* < 0.001. ** *p* < 0.01. * *p* < 0.05. Major diagonals (in bold) show the square roots of AVEs.

**Table 4:** Impacts of psychological factors and moderating effects of demographic factors.

	Model 1	Model 2	Model 3	Model 4
Intercept	3.276	0.537	0.151	0.127
**Control variables**				
Gender	0.132 **	0.104 **	0.063 *	0.068 *
Age	−0.002	−0.013	0.007	0.005
Education	0.018	−0.068 **	−0.058 **	−0.051 **
Personal income	0.100 ***	0.043 **	0.012	0.015
Number of private cars owned by a family	−0.015	0.014	0.022	0.018
Types of cities	0.045	0.020	0.004	0.001
**Independent variables**				
PU		0.567 ***	0.284 ***	0.278 ***
PEU		0.260 ***	0.147 ***	0.141 ***
PP			0.512 ***	0.524 ***
**Interaction effects**				
GEN × PU				−0.125 **
GEN × PEU				−0.034
GEN × PP				−0.117 **
AGE × PU				0.027
AGE × PEU				0.006
AGE × PP				−0.010
EDU × PU				−0.027
EDU × PEU				−0.037
EDU × PP				0.082 **
INC × PU				0.056 *
INC × PEU				−0.010
INC × PP				−0.020
NUM × PU				−0.010
NUM × PEU				0.033
NUM × PP				−0.052
TYP × PU				−0.008
TYP × PEU				0.022
TYP × PP				−0.018
R^2^	0.016	0.470	0.613	0.622
Adjusted R^2^	0.012	0.467	0.611	0.617
F-value	4.830 ***	780.322 ***	672.219 ***	2.498 ***

Note: *** *p* < 0.001, ** *p* < 0.01, * *p* < 0.05.

**Table 5:** Impacts of policy measures and moderating effects of demographic factors.

	Model 1	Model 2	Model 3
Intercept	3.276	0.331	0.319
**Control variables**			
Gender	0.132 **	0.104 **	0.108 **
Age	−0.002	0.013	0.013
Education	0.018	−0.023	−0.023
Personal income	0.100 ***	0.077 ***	0.072 **
Number of private cars owned by a family	−0.015	−0.019	−0.018
Types of cities	0.045 *	0.035	0.037
**Independent variables**			
FI		0.211 ***	0.216 ***
ID		0.343 ***	0.348 ***
CO		0.117 ***	0.121 ***
LN		0.123 ***	0.113 ***
**Interaction effects**			
GEN × FI			0.047
GEN × ID			−0.030
GEN × CO			−0.055
GEN × LN			0.041
AGE × FI			−0.026
AGE × ID			0.011
AGE × CO			−0.020
AGE × LN			−0.001
EDU × FI			0.041
EDU × ID			0.015
EDU × CO			−0.001
EDU × LN			0.092 **
INC × FI			−0.053 *
INC × ID			0.051
INC × CO			0.015
INC × LN			0.037
NUM × FI			−0.031
NUM × ID			−0.065 *
NUM × CO			0.019
NUM × LN			0.035
TYP × FI			0.053 *
TYP × ID			−0.001
TYP × CO			−0.069 *
TYP × LN			0.025
R^2^	0.016	0.327	0.336
Adjusted R^2^	0.012	0.323	0.324
F-value	4.830 ***	210.015 ***	2.691 **

Note: *** *p* < 0.001, ** *p* < 0.01, * *p* < 0.05.

## Data Availability

Some or all data, models, or code generated or used during the study are available from the corresponding author by request.

## Appendix A

**Table A1:** Constructs and measurement items.

Constructs	Code	Measurement Items
TAM-related factors (adapted from Herrenkind et al. [32,33] and Jing et al. [34])		
Adoption intention	AI1	If I have a chance, I would adopt AVs frequently.
	AI2	If it is affordable, I would buy an AV.
	AI3	If I have a choice, I would adopt an AV instead of a traditional vehicle.
Perceived usefulness	PU1	Using AVs will save my time.
	PU2	Using AVs will increase my productivity when moving around. (×)
	PU3	Using AVs will be helpful in my daily life.
Perceived ease of use	PEU1	It would be easy for me to be skillful at operating an AV.
	PEU2	I could pick up how to operate an AV quite quickly.
	PEU3	It would be difficult for me to learn how to operate an AV.*
Perceived playfulness	PP1	Using AVs will be entertaining.
	PP2	It will be playful to use AVs.
	PP3	Many aspects of AVs intrigue me. (×)
Policy measures (adapted from Wang et al. [19]; Imai [21]; Shabanpour et al. [49]; and Chen et al. [50])		
Financial incentivization	FI1	Direct subsidy policy will appeal to me to buy AVs. (about CNY 60,000 to 90,000)
	FI2	Exemption from road tolling will be important to me to use AVs.
	FI3	Exemption from purchase tax and value-added tax will be valuable to me to buy AVs.
Information dissemination	ID1	Publicity for AVs energy conservation and emission reduction will be important to me to use AVs. (×)
	ID2	Information on AVs safety and reliability will be useful to me to use AVs.
	ID3	Advertising on AVs travel ease and efficiency will be helpful to me to adopt AVs.
Convenience	CO1	AVs are permitted to own a dedicated lane will be useful to me to use AVs.
	CO2	AVs are allowed to operate at a higher speed than traditional cars will be attractive to me to use AVs. (×)
	CO3	AVs are unrestricted by the traffic regulations (e.g., even-and-odd license plate numbers) will be helpful to me to adopt AVs.
Legal normalization	LN1	Improving the application of laws related to AVs will be important to me to adopt AVs.
	LN2	Improving the insurance related to AVs will be useful to me to adopt AVs.
	LN3	Improving the governance to AVs will be helpful to me to adopt AVs.

Note: × suggests that the items were removed due to low factor loadings, and * suggests that it is reverse-scored.

## References

[1] Möller T., Padhi A., Pinner D., Tschiesner A. (2019). The Future of Mobility Is at Our Doorstep.

[2] Yuen K.F., Wong Y.D., Ma F., Wang X. (2020). The determinants of public acceptance of autonomous vehicles: An innovation diffusion perspective. J. Clean. Prod..

[3] Fagnant D.J., Kockelman K. (2015). Preparing a nation for autonomous vehicles: Opportunities, barriers and policy recommendations. Transp. Res. Part A Policy Pract..

[4] Becker F., Axhausen K.W. (2017). Literature review on surveys investigating the acceptance of automated vehicles. Transportation.

[5] Wang J., Peeta S., He X. (2019). Multiclass traffic assignment model for mixed traffic flow of human-driven vehicles and connected and autonomous vehicles. Transp. Res. Part B Methodol..

[6] Massar M., Reza I., Rahman S.M., Abdullah S.M.H., Jamal A., Al-Ismail F.S. (2021). Impacts of autonomous vehicles on greenhouse gas emissions—Positive or negative?. Int. J. Environ. Res. Public Health.

[7] Narayanan S., Chaniotakis E., Antoniou C. (2020). Shared autonomous vehicle services: A comprehensive review. Transp. Res. Part C Emerg. Technol..

[8] Haboucha C.J., Ishaq R., Shiftan Y. (2017). User preferences regarding autonomous vehicles. Transp. Res. Part C Emerg. Technol..

[9] Panagiotopoulos I., Dimitrakopoulos G. (2018). An empirical investigation on consumers’ intention towards autonomous driving. Transp. Res. Part C Emerg. Technol..

[10] Dirsehan T., Can C. (2020). Examination of trust and sustainability concerns in autonomous vehicle adoption. Technol. Soc..

[11] Yuen K.F., Cai L., Qi G., Wang X. (2021). Factors influencing autonomous vehicle adoption: An application of the technology acceptance model and innovation diffusion theory. Technol. Anal. Strateg. Manag..

[12] Guo Y., Souders D., Labi S., Peeta S., Benedyk I., Li Y. (2021). Paving the way for autonomous Vehicles: Understanding autonomous vehicle adoption and vehicle fuel choice under user heterogeneity. Transp. Res. Part A Policy Pract..

[13] Potoglou D., Kanaroglou P.S. (2007). Household demand and willingness to pay for clean vehicles. Transp. Res. Part D Transp. Environ..

[14] Coad A., De Haan P., Woersdorfer J.S. (2009). Consumer support for environmental policies: An application to purchases of green cars. Ecol. Econ..

[15] Li W., Long R., Chen H. (2016). Consumers’ evaluation of national new energy vehicle policy in China: An analysis based on a four paradigm model. Energy Policy.

[16] Wang S., Fan J., Zhao D., Yang S., Fu Y. (2016). Predicting consumers’ intention to adopt hybrid electric vehicles: Using an extended version of the theory of planned behavior model. Transportation.

[17] Helveston J.P., Liu Y., Feit M.D., Fuchs E., Michalek J.J. (2015). Will subsidies drive electric vehicle adoption? Measuring consumer preferences in the U.S. and China. Transp. Res. Part A Policy Pract..

[18] Wang N., Pan H., Zheng W. (2017). Assessment of the incentives on electric vehicle promotion in China. Transp. Res. Part A Policy Pract..

[19] Wang S., Li J., Zhao D. (2017). The impact of policy measures on consumer intention to adopt electric vehicles: Evidence from China. Transp. Res. Part A Policy Pract..

[20] Maurer M., Gerdes J.C., Lenz B., Winner H. (2016). Autonomous Driving: Technical, Legal and Social Aspects.

[21] Imai T. (2019). Legal regulation of autonomous driving technology: Current conditions and issues in Japan. IATSS Res..

[22] Hulse L.M., Xie H., Galea E.R. (2018). Perceptions of autonomous vehicles: Relationships with road users, risk, gender and age. Saf. Sci..

[23] Hudson J., Orviska M., Hunady J. (2019). People’s attitudes to autonomous vehicles. Transp. Res. Part A Policy Pract..

[24] Davis F.D. (1989). Perceived usefulness, perceived ease of use, and user acceptance of information technology. MIS Q..

[25] Fayad R., Paper D. (2015). The technology acceptance model e-commerce extension: A conceptual framework. Procedia Econ. Financ..

[26] Al-Emran M., Mezhuyev V., Kamaludin A. (2018). Technology Acceptance Model in M-learning context: A systematic review. Comput. Educ..

[27] Taherdoost H. (2018). Development of an adoption model to assess user acceptance of e-service technology: E-Service Technology Acceptance Model. Behav. Inf. Technol..

[28] Koul S., Eydgahi A. (2018). Utilizing technology acceptance model (TAM) for driverless car technology adoption. J. Technol. Manag. Innov..

[29] Kaur K., Rampersad G. (2018). Trust in driverless cars: Investigating key factors influencing the adoption of driverless cars. J. Eng. Technol. Manag..

[30] Jing P., Huang H., Ran B., Zhan F., Shi Y. (2019). Exploring the factors affecting mode choice Intention of autonomous vehicle based on an extended theory of planned behavior—A case study in China. Sustainability.

[31] Lee J., Lee D., Park Y., Lee S., Ha T. (2019). Autonomous vehicles can be shared, but a feeling of ownership is important: Examination of the influential factors for intention to use autonomous vehicles. Transp. Res. Part C Emerg. Technol..

[32] Herrenkind B., Brendel A.B., Nastjuk I., Greve M., Kolbe L.M. (2019). Investigating end-user acceptance of autonomous electric buses to accelerate diffusion. Transp. Res. Part D Transp. Environ..

[33] Herrenkind B., Nastjuk I., Brendel A.B., Trang S., Kolbe L.M. (2019). Young people’s travel behavior-Using the life-oriented approach to understand the acceptance of autonomous driving. Transp. Res. Part D Transp. Environ..

[34] Jing P., Xu G., Chen Y., Shi Y., Zhan F. (2020). The determinants behind the acceptance of autonomous vehicles: A systematic review. Sustainability.

[35] Bruner G.C., Kumar A. (2005). Explaining consumer acceptance of handheld Internet devices. J. Bus. Res..

[36] Nysveen H., Pedersen P.E., Thorbjornsen H. (2005). Explaining intention to use mobile chat services: Moderating effects of gender. J. Consum. Mark..

[37] Wu J.H., Wang S.C. (2005). What drives mobile commerce?: An empirical evaluation of the revised technology acceptance model. Inf. Manag..

[38] Ahn T., Ryu S., Han I. (2007). The impact of Web quality and playfulness on user acceptance of online retailing. Inf. Manag..

[39] Hur H.J., Lee H.K., Choo H.J. (2017). Understanding usage intention in innovative mobile app service: Comparison between millennial and mature consumers. Comput. Hum. Behav..

[40] Moon J.W., Kim Y.G. (2001). Extending the TAM for a World-Wide-Web context. Inf. Manag..

[41] Bonges H.A.I., Lusk A.C. (2016). Addressing electric vehicle (EV) sales and range anxiety through parking layout, policy and regulation. Transp. Res. Part A Policy Pract..

[42] Egbue O., Long S. (2012). Barriers to widespread adoption of electric vehicles: An analysis of consumer attitudes and perceptions. Energy Policy.

[43] Zhang X., Wang K., Hao Y., Fan J.L., Wei Y.M. (2013). The impact of government policy on preference for NEVs: The evidence from China. Energy Policy.

[44] Gong H., Wang M.Q., Wang H. (2013). New energy vehicles in China: Policies, demonstration, and progress. Mitig. Adapt. Strateg. Glob. Chang..

[45] White L.V., Sintov N.D. (2017). You are what you drive: Environmentalist and social innovator symbolism drives electric vehicle adoption intention. Transp. Res. Part A Policy Pract..

[46] Klöckner C.A., Nayum A., Mehmetoglu M. (2013). Positive and negative spillover effects from electric car purchase to car use. Transp. Res. Part D Transp. Environ..

[47] Howley M., Dennehy E., O’Gallachoir B. (2009). Energy in Transport: 2009 Report.

[48] Li S., Liu Y., Wang J. Factors Affecting the Electric Vehicle Demonstration: 14 International Cities/Regions Cases. Proceedings of the 2015 International Conference on Logistics, Informatics and Service Sciences (LISS).

[49] Shabanpour R., Golshani N., Shamshiripour A., Mohammadian A.K. (2018). Eliciting preferences for adoption of fully automated vehicles using best-worst analysis. Transp. Res. Part C Emerg. Technol..

[50] Chen S., Wang H., Meng Q. (2019). Designing autonomous vehicle incentive program with uncertain vehicle purchase price. Transp. Res. Part C Emerg. Technol..

[51] Blamey R.K., Bennett J.W., Louviere J.J., Morrison M.D., Rolfe J. (2000). A test of policy labels in environmental choice modeling studies. Ecol. Econ..

[52] Wang Q. Consumer adoption of new technological products—Implications for take-up of low carbon technologies. Proceedings of the Conference on Challenges in the Transition to a Low Carbon Society, Warwick Business School.

[53] Graham-Rowe E., Gardner B., Abraham C., Skippon S., Dittmar H., Hutchins R., Stannard J. (2012). Mainstream consumers driving plug-in battery-electric and plug-in hybrid electric cars: A qualitative analysis of responses and evaluations. Transp. Res. Part A Policy Pract..

[54] Wallis N., Lane B., Consultancy E.T. (2013). Electric Vehicles: Improving Consumer Information to Encourage Adoption.

[55] She Z.Y., Sun Q., Ma J.J., Xie B.C. (2017). What are the barriers to widespread adoption of battery electric vehicles? A survey of public perception in Tianjin, China. Transp. Policy.

[56] Tang S., Lo H.K. (2008). The impact of public transport policy on the viability and sustainability of mass railway transit-The Hong Kong experience. Transp. Res. Part A Policy Pract..

[57] Zhang X., Zhang Q., Sun T., Zou Y., Chen H. (2018). Evaluation of urban public transport priority performance based on the improved TOPSIS method: A case study of Wuhan. Sustain. Cities Soc..

[58] Sun L., Huang Y., Liu S., Chen Y., Yao L., Kashyap A. (2017). A completive survey study on the feasibility and adaptation of EVs in Beijing, China. Appl. Energy.

[59] Varian H.R. (1992). Microeconomics Analysis.

[60] Nunnally J.C., Bernstein I.H. (1994). Psychometric Theory.

[61] Hair J.F., Black W.C., Babin B.J., Anderson R.E., Tatham R.L. (2006). Multivariate Data Analysis.

[62] Fornell C., Larcker D.F. (1981). Evaluating structural equation models with unobservable variables and measurement error. J. Mark. Res..

[63] König M., Neumayr L. (2017). Users’ resistance towards radical innovations: The case of the self-driving car. Transp. Res. Part F Traffic Psychol. Behav..

[64] Liljamo T., Liimatainen H., Pollanen M. (2018). Attitudes and concerns on automated vehicles. Transp. Res. Part F Traffic Psychol. Behav..

[65] Nordhoff S., Van Arem B., Happee R. (2016). Conceptual model to explain, predict, and improve user acceptance of driverless podlike vehicles. Transp. Res. Rec..

